# Impact of a goal directed fluid therapy algorithm on postoperative morbidity in patients undergoing open right hepatectomy: a single centre retrospective observational study

**DOI:** 10.1186/s12871-019-0803-x

**Published:** 2019-07-31

**Authors:** Laurence Weinberg, Lois Mackley, Alexander Ho, Steven Mcguigan, Damian Ianno, Matthew Yii, Jonathan Banting, Vijayragavan Muralidharan, Chong Oon Tan, Mehrdad Nikfarjam, Chris Christophi

**Affiliations:** 1 Department of Anesthesia, Austin Health, Heidelberg, Victoria Australia; 2 Department of Surgery, Austin Health, University of Melbourne, Heidelberg, Victoria Australia

**Keywords:** Abdominal surgery, Hepatectomy, Right hepatectomy, Monitoring, Fluid therapy, Surgery, Goal directed therapy

## Abstract

**Background:**

Right hepatectomy is a complex procedure that carries inherent risks of perioperative morbidity. To evaluate outcome differences between a low central venous pressure fluid intervention strategy and a goal directed fluid therapy (GDFT) cardiac output algorithm we performed a retrospective observational study. We hypothesized that a GDFT protocol would result in less intraoperative fluid administration, reduced complications and a shorter length of hospital stay.

**Methods:**

Patients undergoing hepatectomy using an established enhanced recovery after surgery (ERAS) programme between 2010 and 2017 were extracted from a prospectively managed electronic hospital database. Inclusion criteria included adult patients, undergoing open right (segments V-VIII) or extended right (segments IV-VIII) hepatectomy. Primary outcome: amount of intraoperative fluid administration used between the two groups. Secondary outcomes: type and amount of vasoactive medications used, the development of predefined postoperative complications, hospital length of stay, and 30-day mortality. Complications were defined by the European Perioperative Clinical Outcome definitions and graded according to Clavien-Dindo classification. The association between GDFT and the amount of fluid and vasoactive medication used was investigated using logistic and linear regression models.

**Results:**

Fifty-eight consecutive patients were identified. 26 patients received GDFT and 32 received Usual care. There were no significant differences in baseline patient characteristics. Less intraoperative fluid was used in the GDFT group: median (IQR) 2000 ml (1175 to 2700) vs. 2750 ml (2000 to 4000) in the Usual care group; *p* = 0.03. There were no significant differences in the use of vasoactive medications. Postoperative complications were similar: 9 patients (35%) in the GDFT group vs. 18 patients (56%) in the Usual care group; *p* = 0.10, OR: 0.41; (95%CI: 0.14 to 1.20). Median (IQR) length of stay for patients in the GDFT group was 7 days (6:8) vs. 9 days (7:13) in the Usual care group; incident rate ratio 0.72 (95%CI: 0.56 to 0.93); *p* = 0.012. There was no difference in perioperative mortality.

**Conclusions:**

In patients undergoing open right hepatectomy with an established ERAS programme, use of GDFT was associated with less intraoperative fluid administration and reduced hospital length of stay when compared to Usual care. There were no significant differences in postoperative complications or mortality.

**Trial registration:**

Australian New Zealand Clinical Trials Registry: no12619000558123 on 10/4/19.

## Introduction

Right hepatectomy is a complex procedure involving removal of a significant amount of liver parenchymal tissue. Despite technical advances in surgery, anesthesia and critical care medicine, approximately 20–30% of patients undergoing right hepatectomy will have a significant complication. Reported complications include massive hemorrhage and associated hypotension, acute kidney injury, perioperative liver and respiratory failure, bile leak, intra-abdominal infection, and systemic sepsis [1]. In high-volume hepatobiliary centres the reported mortality after major hepatic resection varies between 0 and 6% [1].

Over the last two decades enhanced recovery after surgery (ERAS) programmes for liver resection have reported a number of beneficial outcomes including reduced overall morbidity and hospital length of stay [2–4]. Anesthesia for major liver resection has traditionally concentrated on a “restrictive” fluid therapy approach i.e. “low central venous pressure (CVP) anesthesia”, which is applied for fluid intervention during the hepatic dissection and resection phases of surgery. A meta-analysis has recently questioned this practice reporting that low CVP anesthesia for liver resection is not associated with improvement in postoperative morbidity or length of hospital stay [5] when compared to normal CVP anesthesia.

**Fig. 1:**
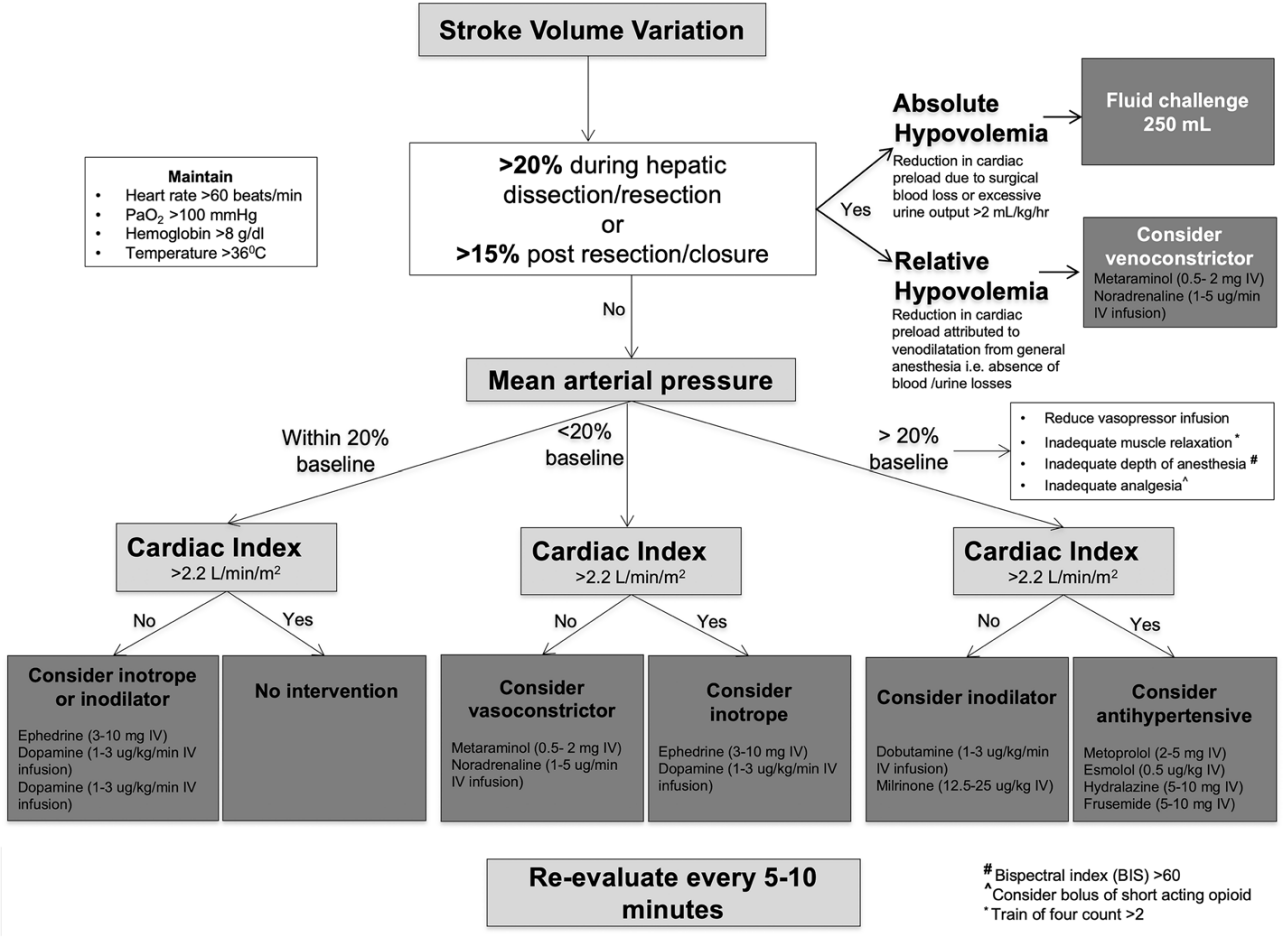
Goal directed fluid therapy cardiac output-guided algorithm

At our institution, we implemented a surgery-specific, cardiac output-guided algorithm to optimize fluid therapy and oxygen delivery for patients undergoing pancreatic surgery [6, 7]. A surgery-specific modification of the algorithm was developed for patients undergoing complex liver resection surgery (Fig. 1). Therefore, to evaluate differences between our “traditional” fluid intervention strategy and our goal directed therapy (GDFT) algorithm in the amount intraoperative fluid and vasoactive medications administered during surgery, the development of postoperative complications, and hospital length of stay, we performed a retrospective observational study comparing patients who underwent right hepatectomy using “usual care” i.e. fluid restriction and low CVP anesthesia during hepatic resection and dissection, and compared their outcomes to patients undergoing right hepatectomy using a GDFT approach i.e. a surgery-specific cardiac output-guided algorithm as outlined in Fig. 1. We hypothesized that for patients undergoing open right hepatectomy, compared to usual care, GDFT using a cardiac output-guided algorithm results in less intraoperative fluid administration, reduced postoperative complications, and improved length of hospital stay.

## Methods

The study was approved by Austin Health Human Research Ethics Committee (HREC no: LNR/2015/Austin/321). As this was a retrospective study, the need for informed participant consent was waived by the Ethics Committee. The study was registered with the Australian New Zealand Clinical Trials Registry (ACTR number: 12619000558123). The study was undertaken at Austin hospital, a university teaching hospital with a high-volume case load in hepatobiliary and pancreatic surgery, including liver transplantation. All patients underwent a standardized ERAS liver protocol that included preoperative optimization of medical comorbidities, optimization of hemoglobin and glycemic control, and early postoperative mobilization as part of an ERAS programme. A summary of the ERAS protocol is presented in Table 1. ERAS for major liver resection was introduced at our institution in August 2009 and by 2010 was established as standard care. Advanced hemodynamic monitoring was not used routinely by all anesthesiologists as a part of the liver ERAS programme, with some anesthesiologists using a “low CVP approach” for intraoperative fluid intervention.

**Table 1:** Enhanced recovery after surgery protocol for open liver resection

Preoperative	- Preoperative multidisciplinary evaluation
	- Cardiac risk stratification with transthoracic echocardiography and stress thallium if clinically indicated
	- Optimization of medical comorbidities
	- Education of patients and families and informed consent
	- Urea, creatinine, electrolytes, estimated glomerular filtration rate, albumin, liver function tests, ferritin, hemoglobin, platelet white cell count, prothrombin time, fasting glucose, activated partial thromboplastin time, fibrinogen, chest x-ray, EKG
Intraoperative	Day of surgery
	- Two hours fasting for clear fluids, 6 h fasting for light meal
	Anesthesia protocol
	- No preoperative IV fluid loading
	- Spinal analgesia: intrathecal morphine (200-400μg)
	- Induction: propofol (1 mg/kg), fentanyl (3 μg/kg)
	- Maintenance: volatile or propofol infusion (BIS of 40–60)
	- Intraoperative analgesia: remifentanil infusion (0.1–0.3 μg/kg/hr)
	- Prophylactic thromboembolic prophylaxis (enoxaparin 40 mg SC)
	- Antibiotic prophylaxis (ceftriaxone/ampicillin/metronidazole)
	- Pre-hepatic transection phase: fluid restriction
	- Hepatic transection phase: fluid restriction, low central venous pressure (< 8 mmHg) using reverse Trendelenburg positioning and glyceryl trinitrate IVI infusion (5–20 μg/min) if required
	- Post-hepatic transection phase: restoration of euvolemia
	- Paracetamol 1 g IV
	- Removal of nasogastric tube at completion of surgery
Day of surgery and & Postoperative Day 1	Analgesia
	- Patient controlled analgesia with fentanyl or oxycodone
	- Fentanyl infusion (10 μg/hr) IV
	- Ketamine infusion 0.05–0.1 mg/kg//hr. IV
	- Paracetamol 1 g IV/po TDS
	Fluid intervention
	- Oral fluids encouraged and soft diet
	- Balanced crystalloid maintenance therapy: 125 ml/hr
	- Albumex 4% 250 ml boluses at discretion of clinicians
	Other
	- Metoclopramide 15 mg IV TDS
	- Potassium and magnesium supplementation
	- Vitamin K 10 mg daily
	- Continue antibiotics for 24 h
	- Dihydrogen phosphate ions (14.5 mmol IV TDS)
	- Pantoprazole 40 mg IV/po daily
	- Heparin 5000 IU SC BD
	- Physiotherapy: early mobilization within 6 h of surgery
Postoperative Day 2	Analgesia
	- Patient controlled analgesia with fentanyl or oxycodone
	- Ketamine infusion ceased
	- Paracetamol 1 g po TDS
	- Tramadol 50–100 mg IV/po QID prn
	Fluid intervention
	- Oral fluids encouraged and soft diet
	- Maintenance fluid therapy reduced to 83 mls/hr
	Other
	- Metoclopramide 15 mg IV TDS
	- Pantoprazole 40 mg po daily
	- Vitamin K 10 mg daily
	- Dihydrogen phosphate ions (14.5 mmol IV TDS)
	- Potassium and magnesium supplementation
	- Physiotherapy: early mobilization TDS
	- Antithrombotic prophylaxis
	- Urinary catheter removed
	Analgesia
	- Patient Controlled Analgesia with fentanyl or oxycodone
	- Stop ketamine infusion
	- Strict QID paracetamol
	- PRN tramadol
	Fluid intervention
	- Aim for neutral fluid balance
	- Reduce maintenance fluid therapy to 42 mls/hr.
	- Soft ward diet
	Other
	- Removal of central venous catheter
	- Removal of urinary catheter
	- Daily weight
	- Strict metoclopramide 15 mg IV TDS
	- Potassium and magnesium supplementation
	- Pantoprazole 40 mg daily
	- Use of diuretic if positive fluid balance (frusemide 10–20 mg)
	- Vitamin K 10 mg daily
	- Dihydrogen phosphate ions (14.5 mmol IV TDS)
	- Continue antithrombotic prophylaxis
	- Physiotherapy: continue mobilization TDS
Postoperative Day 3 to hospital discharge	Analgesia
	- Patient controlled analgesia ceased
	- Oxycodone (IR) 10 mg 4 hourly prn
	- Paracetamol 1 g prn TDS
	- Tramadol 50–100 mg prn TDS
	Fluid intervention
	- Advance oral diet
	Other
	- Daily weight
	- Potassium and magnesium supplementation
	- Pantoprazole 40 mg PO
	- Continue antithrombotic prophylaxis
	- Coloxyl 100 mg BD
	- Physiotherapy: continue mobilization TDS

For this study we extracted data between 2010 and 2017 from a prospectively managed electronic hospital database for all patients aged > 18 years who underwent liver resection surgery with a standard ERAS protocol. Patients were identified using International Statistical Classification of Diseases (ICD) codes specific to liver resection. ICD codes of the following surgical categories were included: ‘excision of lesion of liver’, ‘segmental resection of liver’, ‘lobectomy of liver’, ‘trisegmental resection of liver’, ‘segmental resection of liver for trauma’, ‘lobectomy of liver for trauma’, and ‘trisegmental resection of liver for trauma’. From this search we then included patients undergoing right hepatectomy (segments V-VIII) or extended right hepatectomy (segments IV-VIII). The principle procedure was confirmed using the detailed operation report. Patients undergoing left hepatectomy, minor resections, non-anatomical segmental resections and wedge resections were excluded. We also excluded patients with atrial fibrillation and significant cardiac arrhythmias (bigeminy, trigeminy, pacemaker dependent) due to a lack of precision of the stroke volume variations in these settings. High-volume (greater than 10 major liver resections per year) hepatobiliary surgeons performed all operations. A dedicated group of 8 hepatobiliary-liver transplant anesthesiologists provided care for all the surgeries.

All patients underwent a preoperative multidisciplinary assessment including optimization of cardiorespiratory status. All patients underwent preoperative hemoglobin optimization based on the National Blood Authority of Australia’s patient blood management initiative [8]. Standard perioperative care included strict transfusion practice in accordance with these guidelines. Postoperatively, all patients were admitted to the intensive care unit for at least one overnight stay, and then discharged to a dedicated hepatobiliary surgical ward under a multidisciplinary team of hepatobiliary surgeon, anesthesiologist, perioperative physician, and pain clinician.

General anesthesia was managed using a liver ERAS protocol designed to standardize care. Invasive monitoring using an arterial line and central venous catheter was employed for all patients. The arterial line was inserted prior to induction of anesthesia. The central venous catheter was inserted after induction of anesthesia but before commencement of surgery. All patients, unless contraindicated received intrathecal morphine analgesia. Epidural analgesia was not employed in any patient. Patients did not receive any intravenous fluid loading prior to induction of anesthesia. There was no maintenance fluid therapy administered. Fluid therapy consisted of either 4% or 20% albumen (CSL Behring, Broadmeadows, Australia), or the balanced crystalloid, PlasmaLyte® solution (Baxter Healthcare, Toongabbie, Australia). Autologous venesection was not used for any patients. Blood and blood products were administered as per standard hospital guidelines as clinically indicated. The perioperative ERAS pathway is summarized in Table 1.

### Fluid intervention and vasoactive medication management: usual care group

Intraoperatively, fluid intervention and use of vasoactive drugs for the Usual care group was at the discretion of the attending anesthesiologist. There was no maintenance fluid therapy administered. Fluid therapy consisted of either 4% or 20% albumen, or the balanced crystalloid, PlasmaLyte. The type and volume of fluid administered was at the discretion of the anesthesiologist. Acute normovolemic hemodilution was not utilized in any of the patients. As part of standard care, central venous pressure was generally maintained at less than 8 mmHg during the pre-hepatic transection and dissection phases. Reverse Trendelenburg position and glyceryl trinitrate (5–20 μg/min) were further employed to decrease CVP below 5 mmHg if the patient’s mean arterial pressure (MAP) was within 20% of the baseline value. After completion of the liver transection, euvolemia was restored with crystalloid or colloid fluid intervention, aiming for a target CVP of between 10 and 12 cmH_2_O. Use of vasoconstrictors e.g. metaraminol, phenylephrine, or noradrenaline was at the discretion of the anesthesiologist to support MAP to within 20% of baseline values.

### Fluid intervention and vasoactive medication management: GDFT group

Patients in the GDFT group received intraoperative advanced hemodynamic monitoring using a FloTrac system (FloTrac System 4.0, Edwards Lifesciences, Irvine, CA, USA). The FloTrac sensor was used with an EV1000 clinical platform to continuously measure and display key flow parameters, including cardiac output, stroke volume and systemic vascular resistance. Intraoperative intravenous fluid and vasoactive medications were given as per a goal directed algorithm with adapted stroke volume variation goals specific to major liver resection outlined in Fig. 1. A stroke volume variation of > 20% during liver dissection or resection and > 15% post resection and during closure was targeted. Use of vasoconstrictors e.g. metaraminol, phenylephrine, or noradrenaline was to support MAP to within 20% of baseline values based on the cardiac output guided algorithm. Bolus fluid therapy was either 4% albumen or the balanced crystalloid solution, PlasmaLyte. For other fluid intervention, either 4% or 20% albumen, or PlasmaLyte was used.

Detailed perioperative patient data was entered into an electronic database. Austin Health utilizes Cerner® electronic medical records which allowed comprehensive electronic data capture and access to patient health information in the perioperative setting. Patient characteristics were recorded. Perioperative fluid balances and fluid administration and use vasoactive medications were collected. Fluid balances were calculated by subtracting total output (urine output, blood loss, loss from drains and vomitus) from total input (all intravenous fluid intervention, parental medications or feeding, oral water intake). Third space losses were not included, as they were considered negligible. Electronic hemodynamic data from the FloTrac® device were also recorded.

Complications were defined as any deviation from the normal postoperative course, guided by the European Perioperative Clinical Outcome (EPCO) definitions [9]. Bile leak was defined as presence of bile in the drainage fluid that persisted on post-operative day 4, and acute pancreatitis, defined as an elevation in serum lipase > 3× normal laboratory reference range. Complications were graded according to Clavien-Dindo Classification [10]. Length of stay was determined by the period from completion of surgery to discharge, excluding days in the hospital-in-the-home unit. Readmission was defined as unplanned readmission to the hospital within a 30-day follow-up period. Mortality was considered when it occurred within 30 days of the index admission. The primary outcome was the amount of intraoperative fluid administration used between the two groups. Secondary outcomes included type and amount of vasoactive medications used, the development of predefined postoperative complications, hospital length of stay and 30-day mortality.

Continuous data was tested for normality and measures of central tendency compared as means (standard deviations, SD) using the Student’s t test for normally distributed variables and as medians (interquartile range, IQR) using the Mann-Whitney U test, unless otherwise stated. For the primary end point, the association between GDFT and the amount of fluid used was investigated using linear regression with robust standard error estimation with corresponding effects reported as the difference in means with 95%CI. The association between GDFT and the use of vasoactive drugs was investigated using two types of regression models: a logistic regression for an individual drug being used or not, and linear regression with robust standard error estimation for the amount of drug administered. In order to preserve Type I error at 0.05 for investigating intravenous fluid and vasoactive drugs outcomes where multiple individual comparisons were being made, a multiplicity-corrected *p*-value of less than 0.01 was considered as statistically significant for individual comparisons.

Multivariable associations between GDFT and length of stay were then investigated using negative binomial regression with length of stay treated as the count of days with corresponding effect size reported as Incidence Rate Ratio (IRR) with 95%CI. IRR indicates a factor change in expected length of stay compared to the reference group, e.g. IRR = 2 means that the expected length of stay is twice as long as that of the reference group. The association between GDFT and number of complications was investigated in the same way. A two-tailed p-value less than 0.05 was considered as statistically significant for both these outcomes. In addition, multivariable associations between GDFT and absence or presence of individual complications were investigated using logistic regression modelling with corresponding effects reported as Odds Ratios (ORs) and 95% confidence interval (CI). Statistical analysis was performed using commercial statistical software STATA/IC v.13. We included the STROBE statement checklist of items for observational studies to report our findings [11].

## Results

**Fig. 2:**
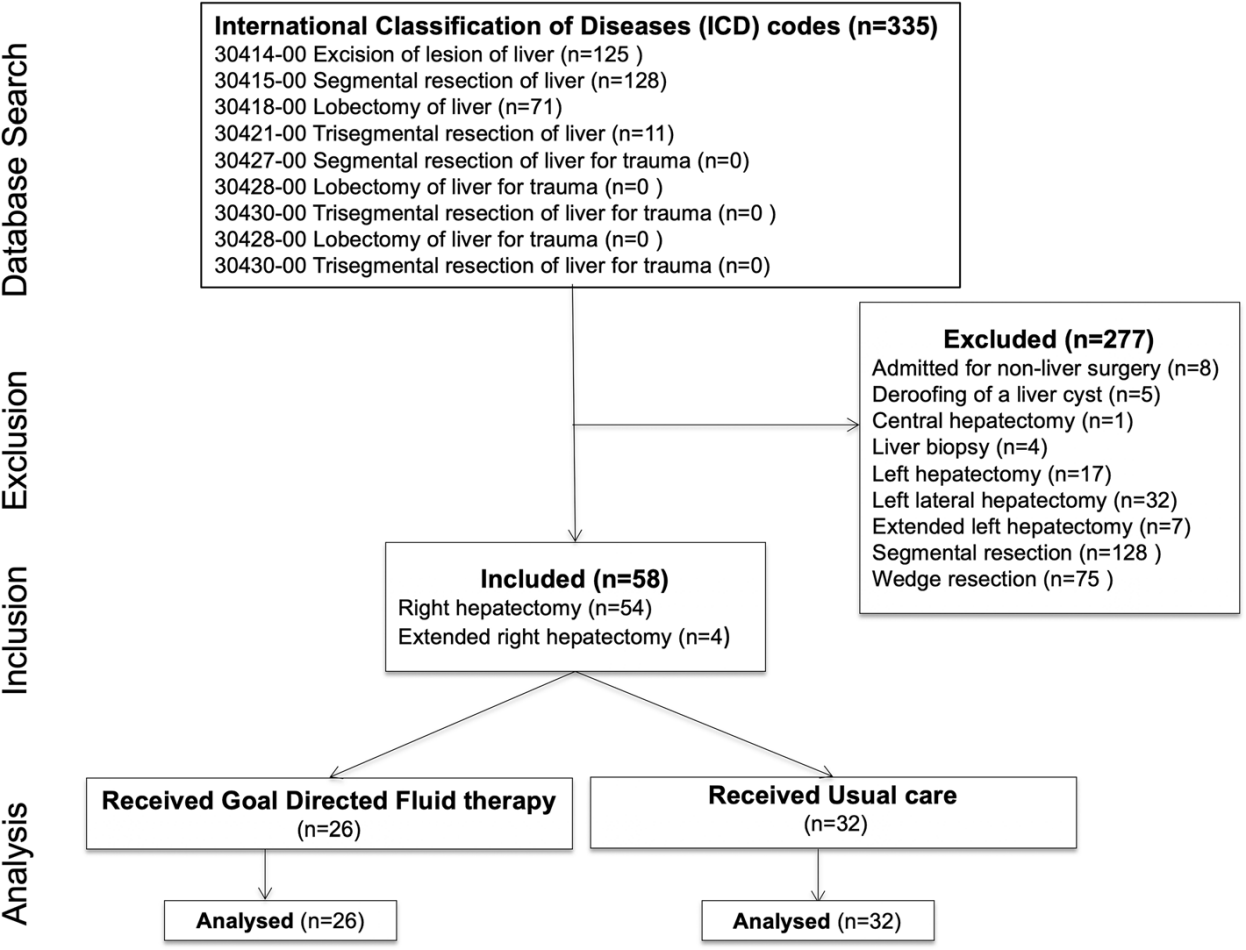
Study flow chart

We identified 335 patients who underwent liver resection at the Austin Hospital between July 2010 and June 2017. Fifty-eight consecutive patients (right hepatectomy, *n* = 54; extended right hepatectomy, *n* = 4) patients were identified for this study. Twenty-six patients received GDFT and 32 patients received Usual care. A flow chart outlining the patient selection process is presented in Fig. 2. There were no significant differences between the groups in baseline characteristics including age, gender and body mass index (Table 2). The median age in the GDFT group and Usual care groups was 66 years (58 to 73) and 66 years (54 to 73) respectively; *p* = 0.42. No statistically significant differences between the groups in gender, body mass index, ASA score and comorbidities were observed (Table 2). Both groups shared similar comorbidity characteristics and all patients were classified as ASA II or III, with the majority of patients ASA class III; 69% GDFT vs. 62% Usual care (*p* = 0.59). Most patients had underlying malignant disease; 92% GDFT vs. 97% Usual care (*p* = 0.58). Preoperative hemoglobin levels, liver function tests, creatinine and coagulation studies were similar with no statistical differences observed between the groups.

**Table 2:** Patient characteristics undergoing open right hepatectomy

	GDFT group (*n* = 26)	Usual care group (*n* = 32)	*p*-value
Patient characteristics			
Age (years)	62 (52 to 71)	62 (52 to 71)	0.68
Male: Female	14 to 12	14 to 18	0.59
Body mass index (kg/m^2^)	25 (23 to 33)	25 (23 to 29)	0.37
ASA Class II	8 (31%)	12 (38%)	0.78
ASA Class III	18 (69%)	20 (62%)	0.78
Malignancy	24 (92%)	31 (97%)	0.58
Preoperative blood tests			
Hemoglobin (g/l)	135 (124 to 146)	131 (119 to 146)	0.75
Albumin (g/L)	42 (39 to 44)	40 (36 to 43)	0.09
Bilirubin (μmol/l)	8.5 (7 to 13.5)	10 (7 to 14)	0.80
Creatinine (μmol/l)	76 (62 to 80)	63 (55 to 81)	0.25
eGFR (mL/min per 1.73 m^2^)	90 (74 to 90)	89 (70 to 90)	0.99
Alkaline phosphatase (IU/L)	122 (65 to 171)	105 (76 to 213)	0.56
Alanine transaminase (U/L)	41 (21 to 71)	28 (19 to 46)	0.09
Platelets (×10^9^/L)	207 (159 to 263)	183 (158 to 273)	0.84
Prothrombin time (s)	11 (11 to 12)	12 (11 to 13)	0.10
Activated partial thromboplastin time (s)	27 (25 to 29)	29 (25 to 33)	0.06
Intraoperative factors			
Volatile anesthesia	4 (15.3%)	5 (16.6%)	> 0.99
Propofol anesthesia	10 (38.5%)	18 (56.3%)	0.30
Volatile and propofol anesthesia	12 (46.2%)	9 (28.1%)	0.18
Duration of surgery (mins)	330 (238 to 356)	305 (246 to 423)	0.76
Specimen weights (g)	800 (465 to 1080)	780 (487 to 1013)	0.98
Lowest temperature (°C)	35.3 (34.9 to 35.6)	35.8 (35.3 to 36.3)	0.004
Baseline central venous pressure	6 (4 to 8)	8 (5 to 10)	0.36
Pre-resection central venous pressure	5 (3 to 7)	4 (2 to 6)	0.12
Post-resection central venous pressure	7 (3 to 10)	5 (2 to 8)	0.15

*ASA* American society of anesthesiologists

Data represented as median (interquartile range) or number (proportion)

### Key outcomes

The median (IQR) length of hospital stay for all patients was 7.5 (6 to 10) days. Median (IQR) length of stay for patients in the GDT group was 7 days (6:8) vs. 9 days (7 to 13) in the Usual care group; IRR 0.72 (95%CI: 0.56 to 0.93**);** *p* = 0.012.

Details of intraoperative variables including fluid intervention are summarized in Table 2. Median (IQR) total intraoperative fluid administration was 2000 ml (1175 to 2700) in the GDFT group compared to 2750 ml (2000 to 4000) in the Usual care group; Effect size: -729 ml (− 1462 to − 3); *p* < 0.03. Median (IQR) crystalloid use was 1250 ml (1000 to 2025) in the GDFT group vs. 2000 ml (1750 to 3150) in the Usual care group; effect size − 799 ml (− 1414 to -86); *p* < 0.007. Correspondingly, median (IQR) intraoperative fluid balances were lower in the GDFT group compared to the Usual care: 1215 ml (613 to 1680) vs. 2095 ml (1330 to 3608), effect size -921 ml (− 1646 to 196), *p* < 0.005. Intraoperative blood loss and hepatic resection weight were similar in both groups (Table 3). The majority of patients in both groups received a vasoactive medication. A summary of vasopressor agent use is summarized in Table 3. Three participants in each group received intraoperative beta-blockers. No participants received any diuretic agent.

**Table 3:** Intraoperative fluid and vasoactive medications in patients undergoing open right hepatectomy

	GDFT group (*n* = 26)	Usual care group (*n* = 32)	Effect Size	*p*-value
Crystalloid therapy				
Number of patients	26 (100%)	32 (100%)		>.99
Total volume (mls)	1250 (1000 to 2025)	2000 (1725 to 3150)	-799 (−1414 to − 186)^b^	0.007
Colloid therapy (excluding blood)				
Number of patients	17 (65%)	18 (56%)		0.59
Total volume (mls)	500 (200 to 1000)	750 (500 to 1000)	−105 (− 173.7 to 382.8)	0.41
Colloid type				
4% albumen (no of patients)	11 (42%)	15 (47%)		0.79
Total volume (mls)	1000 (500:1000)	1000 (500:1000)		
20% albumen (no of patients)	6 (23%)	3 (9%)		0.27
Total volume (mls)	200 (100:200)	200 (100:200)		
Total fluids (including colloids, crystalloids and blood) (mls)	2000 (1175 to 2700)	2750 (2000 to 4000)	−729 (−1462 to −3.03)^a^	0.026
Blood transfusion (number of patients)	3 (11.5%)	2 (6.2%)		0.4
Total volume (ml)	465 (465:1000)	1275 (1275:1400)		
Urine output (ml)	220 (155 to 300)	320 (195 to 485)		0.022
Blood loss (ml)	400 (300 to 950)	400 (300 to 750)	17.0 (−201 to 235)^b^	0.61
Fluid balance	1215 (613 to 1680)	2095 (1330 to 3608)	−921 (−1646 to −196)^b^	0.005
Number of patients receiving a vasoactive medication	20 (77%)	29 (91%)	0.23 (0.04 to 1.1) ^c^	0.12
Metaraminol use (number of patients)	6 (23%)	23 (72%)	0.12 (0.04 to 0.37)^c^	< 0.001
Metaraminol (mg)	0 (0–1)	3 (0–9)	−3.64 (−5.82 to −1.46)^b^	< 0.005
Ephedrine/dopamine use (number of patients)	3 (12%)	8 (25%)	0.39 (0.10 to 1.61)^c^	0.31
Epinephrine (mg)	0 (0–0)	0 (0–8)	4.56 (−9.50 to 0.38)^b^	0.08
Noradrenaline use (number of patients)	15 (46%)	9 (28%)	3.49 (1.23 to 9.47) ^c^	0.03
Noradrenaline (ug)	390 (0 to 870)	0 (0 to 353)	1.67 (− 325 to 662)^b^	0.10

^a^Effect size reported as incidence rate ratio^. b^Effect size reported as difference in means ^c^Effect size reported as odds ratio

Data presented as median (interquartile range), or number (proportion)

Nine patients (35%) developed a postoperative complication in the GDFT group vs. 18 patients (56%) in the Usual care group (OR: 0.41; 95%CI: 0.14 to 1.20; *p* = 0.10). Four patients (15.4%) patients in the GDFT group developed a postoperative kidney injury vs. 1 (3.1%) patient in the Usual care group (*p* = 0.16). The highest median (IQR) creatinine in the first 72 h postoperatively was 71 umol/L (62.0 to 92.7) in the GDFT group vs. 67 umol/L (55.0 to 75.5) in the Usual care group (*p* = 0.17). The immediate postoperative median (IQR) hemoglobin was 117 g/l (105 to 124) in the GDFT group vs. 109 g/l (98.2 to 126.8) in the Usual care group (*p* = 0.34). There were no mortalities in the GDFT group and two mortalities (6.2%) in the Usual care group from postoperative liver failure. There were no readmissions in either group. A summary of postoperative complications and their severity is presented in Table 4.

**Table 4:** Postoperative complications

	GDT group (*n* = 26)	Usual care group (*n* = 32)	*p*-value
Patients with complications	9 (35%)	18 (56%)	0.10
Number of Complications	26	37	Not estimable
Clavien-Dindo Classification (worst complication			
Grade I & II	5 (19.2%)	14 (43.8%)	0.06
Grade III	0	1 (3.1%)	> 0.99
Grade IV	3 (11.5%)	1 (3.1%)	0.31
Grade V	0	2 (6.2%)	0.49
Wound infection			0.44
Superficial surgical site infection	0	0	
Deep surgical site infection	2 (7.7%)	5 (15.6%)	
Sepsis	2 (7.7%)	4 (12.5%)	0.68
Electrolyte abnormality requiring treatment	4 (15.4%)	2 (6.2%)	0.39
Delayed gastric emptying	2 (7.7%)	4 (12.5%)	0.68
Bile leak	1 (3.8%)	2 (6.2%)	> 0.99
Acute kidney injury	4 (15.4%)	1 (3.1%)	0.16
Pulmonary embolus	3 (11.5%)	0	0.08
Pulmonary atelectasis/effusion	2 (7.7%)	3 (9.4%)	> 0.99
Myocardial infarction	0 (0%)	1 (3.1%)	> 0.99
Arrhythmia	0	2 (6.2%)	0.49
Postoperative delirium	3 (11.5%)	1 (3.1%)	0.31
Acute liver failure	0	2 (6.2%)	0.49
Other^a^	2 (7.7%)	5 (15.6%)	0.44
Return to theatre	1 (3.8%)	2 (6.2%)	> 0.99
Death within 30 days	0 (0%)	2 (6.2%)	0.49

^a^ Other: blood transfusion, medical emergency team activation for hypotension, pneumothorax

Data presented as number (proportion)

**Fig. 3:**
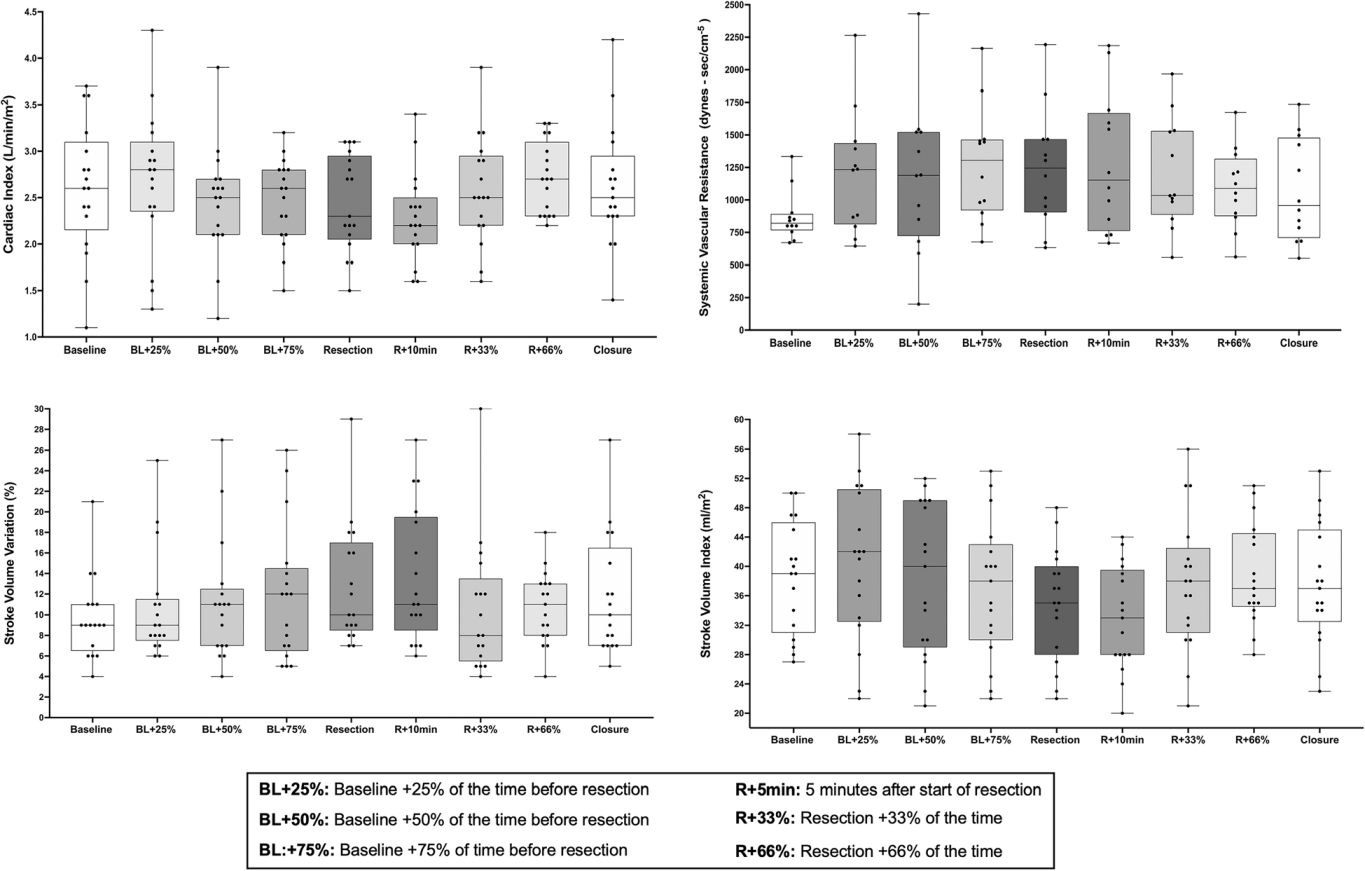
Box and Whisker plots showing presenting intraoperative hemodynamic variables in Goal Directed Fluid Therapy group. Whiskers are maximum and minimum values

**Fig. 4:**
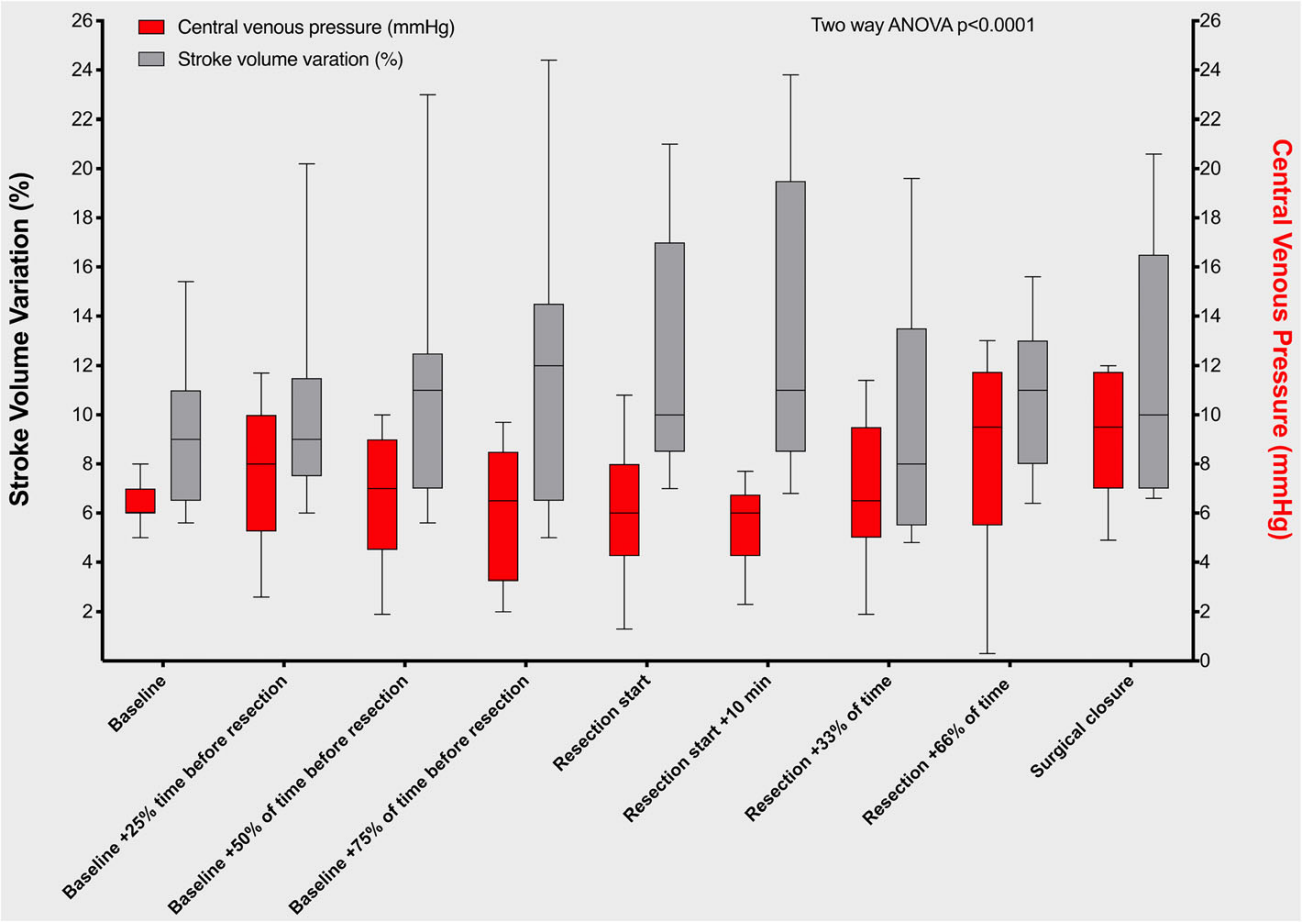
Box and Whiskers plot of central venous pressure and stroke volume variation throughout surgery. Whiskers presented as 10-90th centile

Median (IQR) duration of surgery was 330 min (238 to 356) in the GDFT group and 305 min (246 to 423) in the Usual care group; *p* = 0.76. The median (IQR) tidal volume in the GDFT group was 8 ml/kg (7.1 to 8.8) vs. 7.9 ml/kg (7.3 to 8.8) in the Usual care group (*p* = 0.83). Baseline median (IQR) central venous pressure was 6 cmH_2_0 (4 to 8) in the GDFT group vs. 8 cmH_2_0 (5 to 10) in the Usual care group; *p* = 0.36. The pre-hepatic and post-hepatic resection central venous pressures were 5 cmH_2_0 (3 to 7) and 7 cmH_2_0 (3 to 10), respectively in the GDFT group vs. 4 (2 to 6) cmH_2_0 and 5 (2 to 8) cmH_2_0 in the Usual care group (*p* = 0.12, *p* = 0.15 respectively). The intraoperative cardiac and stroke volume index, central venous pressure, stroke volume variation, and systemic vascular resistance for patients in the GDFT group are presented in Fig. 3. Hemodynamic data from the Flotrac device was missing for 9 patients in the GDFT group; for the remaining 17 patients, the relationship between SVV and CVP for patients in the GDFT group is presented in Fig. 4. Across all time points, two-way repeated ANOVA showed a mean CVP of 6.9 mmHg and a stroke volume variation of 11.4% (Effect size − 4.5, standard error of difference 0.59, 95%CI: − 5.7 to − 3.4, *p* < 0.0001) This inverse relationship was most marked at the start of the hepatic resection and during the first 10 min of the resection (Fig. 4).

Postoperative Day 1 fluid balances were similar in both groups: 1682 ml (1357 to 2229) in the GDFT group vs. 1382 ml (300 to 2195) in the Usual care group; *p* = 0.15. Both groups received a similar amount of fluid on Day 1; 2635 ml (1949 to 3102) in the GDFT group vs. 2348 ml (1713 to 3288) in the Usual care group; *p* = 0.45; a higher urine output was observed in the Usual care group; 1320 ml (965 to 1815) vs. 1000 ml (668 to 1288) in the GDFT group; *p* = 0.04. The median (IQR) length of hospital stay for all patients was 7.5 (6 to 10) days.

## Discussion

We performed a single centre retrospective study investigating the effect of a hemodynamic goal directed algorithm in patients undergoing open right hepatectomy. As hypothesized, we observed that a surgery-specific, patient-specific cardiac output algorithm was associated with restrictive intraoperative fluid management and reduced hospital length of stay when compared to usual care. There were no significant differences observed in perioperative complications or postoperative mortality. The use of a surgery-specific, patient-specific cardiac output algorithm as part of a standard ERAS protocol was safe and our findings have implications for similar algorithms to be tested in prospective clinical trials.

The accuracy of stroke volume variation has been reported to be beneficial in guiding fluid management during hepatic resection [12], with excellent correlation reported with the patient’s volemic status [13, 14]. In our institution, for complex pancreatic and liver resection surgery, we consider a SVV of greater than 20% as a conclusive and clear cut off for effective volume expansion during the hepatic and dissection phases of surgery. This practice stems from research showing that a SVV value between 9 and 13% can be an inaccurate predictor of fluid responsiveness in approximately 25% of patients during general anesthesia [15, 16]. In our institution targeting a SVV threshold of greater than 20% has resulted in improved patient outcomes after pancreaticoduodenectomy [6, 7, 15, 16]. Whilst stroke volume variation monitoring in liver surgery has also been reported to reduce intraoperative bleeding compared to traditional CVP monitoring [17], these findings were not observed in our study. SVV has also been shown to be a useful indicator of intraoperative blood loss without the monitoring of CVP during hepatic resection under clamping of both the infrahepatic inferior vena cava and the portal triad [18], a finding also not observed in our study.

It has recently been reported that a CVP value of less than 8 mmHg or a SVV greater than 13% is able to achieve a minimal blood loss of 100 mL during parenchyma transaction during a living donor hepatectomy [19]. This contrasts with the findings of other studies that report SVV as a less reliable indicator of low CVP because of the weak correlation between SVV and CVP during profound vasodilation states during complex liver surgery [20]. In our study, a low CVP was achieved in both patient cohorts, which may explain why we observed no differences in intraoperative blood loss.

Whilst our findings suggest the use of an advanced hemodynamic cardiac output algorithm in patients undergoing right hepatectomy leads to a more restrictive fluid intervention strategy, the mechanism by which an average reduction of less than one litre of intraoperative fluid could lead to a shorter length of hospital stay remains unclear. Correa-Gallego et al. conducted a prospective randomized trial of patients undergoing liver resection and randomized patients to GDFT using stroke volume variation or to Usual care [21]. Similar to our findings, the GDFT group received less intraoperative fluids, and the incidence of postoperative complications were similar in both groups. In our study, we found that complication rates were not significantly different across the study cohort, and indeed none of the noted complications were directly related to fluid-related complications. Our findings contrast with the other reports that demonstrate fluid optimization guided by SVV during major abdominal surgery is associated with a lower incidence of postoperative organ complications [22]. Of note, we found that hypothermia was common in both our patient groups, and whilst a significantly lower intraoperative temperature was noted in the GDFT group, the clinical significance of this small difference is questionable.

Our study has several key strengths. To date we report a series of patients undergoing open right hepatectomy where a cardiac output-guided hemodynamic algorithm has been used to guide fluid intervention and use of vasoactive therapy. While we cannot establish a causal relationship between this hemodynamic algorithm and improved outcomes, our findings suggest that a cardiac output-guided hemodynamic algorithm is safe and feasible. Use of the algorithm may be applicable to centres that do not employ CVP monitoring as part of routine anesthesia care for major liver resection. Our findings provide pilot data for sample size calculations for future randomized controlled trials in this area.

Our study has some inherent limitations. As this was a retrospective study, we were not able to present data regarding the timing of fluid administration i.e. at which points during the hepatectomy the SVV exceeded 20%. Neither could we investigate the exact correlation between the CVP and SVV throughout surgery as hemodynamic data from 9 patients in the GDFT group were missing. Further, the compliance rate of adherence to the GDFT protocol is unknown. Data regarding postoperative complications was collected from coding on patient’s discharge summaries and then manually cross-checked by two investigators against the medical records to minimize errors in the coding classification of complications. This ensured accuracy of the definitions of complications that were applied across both groups. However, for some complications such as intraoperative blood loss, we could only enter what was documented on the anesthesia chart as weighing of surgical packs for precise measurement of loss blood loss was not accurately recorded for all patients. Whilst GDFT is a technique commonly used by all the anesthesiologists at our institution, it is applied with varying frequencies. We acknowledge that there may be some bias introduced by certain anesthesiologists being more likely to use advanced hemodynamic monitoring as part of their usual practice and/or certain anesthesiologists being more likely to choose GDFT for certain patient groups. We acknowledge this selection bias may have resulted in a Type 1 error.

We did not collect individual clinicians’ outcomes and could not adjust for the impact this may have had for several reasons. Firstly, we were unable to accurately assign each patient to a single surgeon or anesthesiologist, as it is common at our institution for complex cases to be attended by two consultant surgeons. In addition, surgical and anesthesiology advanced trainees frequently assist with varying components of the surgery. Adjusting for surgical or anesthesiology confounding is not possible. Furthermore, certain hepatobiliary surgeons undertake several more complex resections with extensive vascular reconstructions each year, which independently carry increased risks of perioperative morbidity. Therefore, assessing individual surgeons without adjusting for surgical complexity is also unsatisfactory. Finally, we acknowledge that our series of right hepatectomy patients is small. We are unable to extrapolate the findings of our cardiac output guided algorithm to laparoscopic liver surgeries, minor liver resections, sicker, older and morbidly obese patients, or to other types of more complex liver surgeries e.g. those that require venous extracorporeal membrane oxygenation support, or patients undergoing liver transplantation.

The study was performed over a seven-year time period, however during this time although there were no changes to surgical or anesthesia techniques or to the ERAS protocol; neither were there any new training interventions or hospital staffing changes made to the hepatobiliary, anesthesiology, critical care and ward teams caring for all patients during the study period. However, we acknowledge the increasing experience and skills of the anesthesiology and surgical teams over time and together with improvements in pain management, we acknowledge that improvement in outcomes may be innate to a time course bias.

## Conclusions

In patients undergoing open right hepatectomy using an established ERAS liver protocol, the use of a patient-specific, surgery-specific cardiac output hemodynamic algorithm was associated with restrictive intraoperative fluid use and a shorter hospital stay compared to a low CVP fluid intervention strategy. The use of GDFT was not associated with any observed reductions in blood loss or reductions in postoperative complications. Our study provides valuable data to support sample size calculations for further prospective GDFT research for complex hepatobiliary surgery.

## Data Availability

Not available as participants did not consent to sharing individual data. Deidentified data can be requested from the corresponding author.

## Abbreviations

BIS: Bispectral index
CI: Confidence interval
CVP: Central venous pressure
EPCO: European perioperative clinical outcome
ERAS: Enhanced recovery after surgery
GDFT: Goal directed therapy
ICD codes: International statistical classification of diseases codes
IQR: Interquartile range
IRR: Incidence rate ratio
IV: Intravenous
SD: Standard deviations
SVV: Stroke volume variation
